# Western Carpathian mid-Permian Magmatism: Petrographic, geochemical, and geochronological data

**DOI:** 10.1016/j.dib.2021.107026

**Published:** 2021-04-20

**Authors:** Gabriel Villaseñor, Elizabeth J. Catlos, Igor Broska, Milan Kohút, Ľubomír Hraško, Kimberly Aguilera, Thomas M. Etzel, J. Richard Kyle, Daniel F. Stockli

**Affiliations:** a Department of Geological Sciences, Jackson School of Geosciences, The University of Texas at Austin, Austin, TX, United States of America; b School of Earth and Atmospheric Sciences, Department of Geophysics, Georgia Institute of Technology, Atlanta, GA, United States of America; c Earth Science Institute of the Slovak Academy of Sciences, Dúbravská cesta 9, 840 05 Bratislava, Slovakia; d State Geological Institute of Dionýz Štúr, Mlynská dolina 1, 817 04 Bratislava, Slovakia; e ExxonMobil, Parkway Spring, Texas, United States of America

**Keywords:** Zircon, Geochronology, Carpathians, Geochemistry, Slovakia, Granite

## Abstract

This study presents geochemical and geochronological data from rock samples collected from the Western Carpathian mountains, eastern Slovakia. Granite assemblages that intrude the Gemeric and Veporic Superunits were imaged using a petrographic microscope to determine rock textures and their mineral assemblages. Zircon grains from seven individual portions of the Gemeric granites (Hnilec, Betliar, Elisabeth Mine, Poproč plutons) and one from the Veporic unit (Klenovec pluton) were dated using Laser Ablation-Inductively Coupled Plasma-Mass Spectrometry (LA-ICP-MS) and Secondary Ion Mass Spectrometry (SIMS). Eight individual portions of the Gemeric unit's Betliar pluton and seven from the Klenovec granite were analyzed for major and trace elements using Fusion Inductively Coupled Plasma (ICP) and Fusion ICP-mass spectrometry. We also report detrital zircon ages from a radiolarite from the Meliata Unit that overlies blueschist and harzburgite-lizardite serpentinite assemblages near the city of Dobšiná, Slovakia. We applied X-ray Diffraction to a sample from the serpentine rocks, which reveal the presence of lizardite. The data are available for re-use to compare to future analyses of these Permian-age granites found in the Carpathian Mountains or similar Permiam assemblages elsewhere more broadly. Data reported in this article relates to G. Villaseñor, E.J. Catlos, I. Broska, M. Kohút, Ľ. Hraško, K. Aguilera, T.M. Etzel, J.R. Kyle, and D.F. Stockli, Evidence for widespread mid-Permian magmatic activity related to rifting following the Variscan orogeny (Western Carpathians), 2021, Lithos.

## Specifications Table

**Table:** 

Subject	Earth and Planetary Sciences
Specific subject area	Geology
Type of data	Tables, figures
How data were acquired	Rock images were acquired using an Olympus BX51 petrographic microscope fitted with a digital camera and Optiscan imaging software. Bulk rock compositional data for the Betliar granites (*n* = 8) were obtained using Fusion Inductively Coupled Plasma (ICP) spectrometry applied to fused samples using a Perkin–Elmer Sciex ICP-Mass Spectrometer (MS). A similar Fusion ICP-MS approach was taken for the Klenovec granite (*n* = 7), but these were obtained at the Laboratory State Geological Institute of Dionýz Štúr, Spišská Nová Ves, in Slovakia. Laser Ablation-Inductively Coupled Plasma-Mass Spectrometry (LA-ICP-MS) zircon ages were obtained using an Element2 High Resolution (HR)-ICP-MS with an Excimer (192 nm) laser ablation system instrumentation in the Geo-Thermochronometry lab at the University of Texas at Austin (UT Austin). Secondary Ion Mass Spectrometry (SIMS) ages were obtained using a CAMECA IMS 1280-HR at UCLA. X-ray Diffraction (XRD) data was obtained from rock powders using a Bruker D8 Advance in the Dept. of Geological Science's Electron Microbeam facility at UT Austin.
Data format	Raw and Analyzed
Parameters for data collection	Data were collected following standards practiced by the analytical facilities.
Description of data collection	Rock thin sections were imaged using the microscope. Rock chips and powders were sent to analytical facilities to obtain the bulk rock composition and XRD data. Individual zircon grains were dated from the samples using the instruments at UT Austin and UCLA.
Data source location	Institution: The University of Texas at Austin City/Town/Region: Austin/Texas Country: USA Latitude and longitude for collected samples/data: See Table 1 for the analyzed rocks' GPS coordinates.
Data accessibility	Repository name: Texas Scholar Works, University of Texas Libraries Direct URL to data: https://doi.org/10.18738/T8/PFWPNR Instructions for accessing these data: Access is provided at the link.
Related research article	Villaseñor G., Catlos E.J., Broska I. , Kohút M., Hraško Ľ., Aguilera K., Etzel T.M., Kyle J.R., Stockli D.F., Widespread evidence of mid-Permian magmatic activity related to rifting following the Variscan orogeny (Inner Western Carpathians), Lithos (2021) 106083, https://doi.org/10.1016/j.lithos.2021.106083.

## Value of the Data

• Data obtained from the Inner Western Carpathian's Gemeric and Veporic granites provide critical information about their petrologic and tectonic history.
• Gemeric and Veporic granite ages provide insight into the assembly of the Inner Western Carpathian mountains.
• Researchers can compare results to other extensional-related Permian felsic alkaline magmatism exposed from the Western Mediterranean through the Western Carpathians to the Central and Southern Alps.
• The data can be re-used to provide further insights regarding European plate motions from the Permian to Triassic.

## 1 Data Description

We present zircon ages and geochemical data from granite plutons that intrude the Gemeric and Veporic Superunits. We report detrital zircon ages from a radiolarite from the Meliata Unit that overlies blueschist and serpentinite assemblages near the city of Dobšiná, Slovakia. The base of the Meliata Unit in the Dobšiná locality is a highly altered blueschist marble mélange complex, and we present X-ray Diffraction (XRD) data from those rocks to better clarify their mineral chemistry. Sample names and locations are listed in Table 1.

**Table 1:** Sample locations.

Rock unit name	sample number(s)	Latitude	Longitude
Hnilec granite	TT51	N48°49.549′	E 20° 29.211′
Betliar granite	IR19A-E, IR20A-C	N48° 44.16′	E 20° 31.74
Elisabeth Mine granite	TT07	N 48° 45.034′	E 20° 29.641′
Poproč granite	TT49	N 48° 43.374′	E 20° 59.004′
Klenovec granite	TT48, VZ-/40 m, VZ-3/93 m, VZ-4/118 m, VZ-4/142 m, LH-39/04, LH-4/04, LH-72/04	N 48° 32.760′	E 19° 49.620′
Dobšiná Radiolarite	TT08	N 48° 49.633′	E 20° 21.988′
Dobšiná Serpentinite	TT08D, TT08H	N 48° 49.633′	E 20° 21.988′

**Table 2:** Summary of the whole-rock geochemical analysis of samples from the Betliar Pluton.

Analyte Symbol	IR19A	IR19B	IR19C	IR 19D	IR 19E	IR 20A	IR 20B	IR 20C
SiO_2_	72.91	74.41	76.42	75.39	76.06	75.53	73.73	74.62
Al_2_O_3_	13.39	13.73	12.76	13.10	12.91	12.14	14.02	14.50
Fe_2_O_3_(T)	2.220	1.210	1.930	1.970	1.310	1.550	1.620	1.560
MnO	0.033	0.016	0.038	0.021	0.014	0.021	0.050	0.027
MgO	0.300	0.300	0.110	0.470	0.480	0.350	0.070	0.410
CaO	0.410	0.180	0.280	0.260	0.200	0.260	0.400	0.700
Na_2_O	3.11	3.51	3.14	3.08	2.83	2.74	3.56	0.13
K_2_O	5.18	4.19	5.01	3.37	4.36	4.62	4.20	4.81
TiO_2_	0.182	0.061	0.078	0.222	0.077	0.186	0.056	0.037
P_2_O_5_	0.200	0.110	0.160	0.160	0.130	0.220	0.380	0.310
LOI	0.940	1.020	0.710	1.180	1.190	1.180	1.000	2.150

Total	98.9	98.7	100.7	99.2	99.6	98.8	99.1	99.2

*Sc*	3	2	3	6	3	4	7	8
Be	4	4	7	4	3	2	2	2
V	14	6	6	14	6	12	< 5	< 5
Ba	390	129	61	141	154	263	76	304
Sr	40	19	18	17	14	24	94	16
Y	27	19	15	26	13	30	6	9
Zr	103	50	62	111	60	89	44	45
Co	2	< 1	1	< 1	1	< 1	< 1	< 1
Zn	40	< 30	30	40	< 30	< 30	< 30	< 30
Ga	19	19	20	20	18	18	33	24
Ge	2	2	2	2	2	2	4	4
As	8	< 5	14	< 5	< 5	< 5	< 5	< 5
Rb	366	312	426	241	312	395	934	560
Nb	6	5	8	9	6	8	31	56
Sn	21	29	33	42	19	94	27	22
Sb	1	0.7	0.9	1	0.8	2.3	< 0.5	1.3
Cs	13	7.5	18.2	6.9	8.1	9	14.1	7.7
La	20.9	8.2	7.0	48.9	2.3	17.2	3.0	1.1
Ce	44.4	16.6	16.9	99.5	5.2	38.3	6.6	3.1
Pr	5.3	2.15	1.87	11.3	0.94	4.35	0.74	0.42
Nd	19.8	8.1	6.4	39.8	4.6	16.0	2.7	1.7
Sm	4.6	2.3	1.9	7.3	1.8	3.9	0.8	0.8
Eu	0.48	0.15	0.10	0.41	0.13	0.24	0.05	< 0.05
Gd	4.3	2.2	1.8	4.9	2.0	3.9	0.7	1.0
Tb	0.8	0.5	0.4	0.8	0.4	0.8	0.2	0.3
Dy	4.9	3.2	2.8	4.6	2.5	5.5	1.1	1.6
Ho	1.0	0.7	0.6	0.9	0.5	1.1	0.2	0.3
Er	2.9	2.0	1.6	2.6	1.4	3.2	0.6	0.7
Tm	0.46	0.35	0.29	0.43	0.25	0.51	0.12	0.14
Yb	2.9	2.5	2.1	2.9	1.8	3.5	0.8	1.1
Lu	0.4	0.36	0.3	0.4	0.27	0.5	0.12	0.14
Hf	2.9	1.7	2.2	2.9	2.1	2.6	2.5	2.7
Ta	1.5	1.6	1.9	2.7	1.9	2.2	12.5	16.4
W	5	4	3	10	5	11	43	22
Tl	1.5	1.1	1.7	0.7	1.0	1.1	1.4	0.9
Pb	21	7.0	14	< 5	< 5	12	12	< 5
Bi	1.6	0.4	< 0.4	3.6	2.2	0.5	1.4	29.5
Th	14.4	9.7	10.7	13.5	9.9	13	10.3	9.8
U	4.2	3.5	2.7	3.5	2.3	3.2	4.0	5.9

Measured but below detection limits: Cr and Ni <20 ppm, Cu <10 ppm, Mo <2 ppm, Ag <0.5 ppm, In <0.2 ppm.

The next series of figures shows photographs of the granites in hand specimens (Fig. 1) and in petrographic thin section (Fig. 2, Fig. 3, Fig. 4, Fig. 5, Fig. 6). CL images of the dated zircons are presented in Fig. 7, Fig. 8, Fig. 9, Fig. 10, Fig. 11, Fig. 12.

**Fig. 1:**
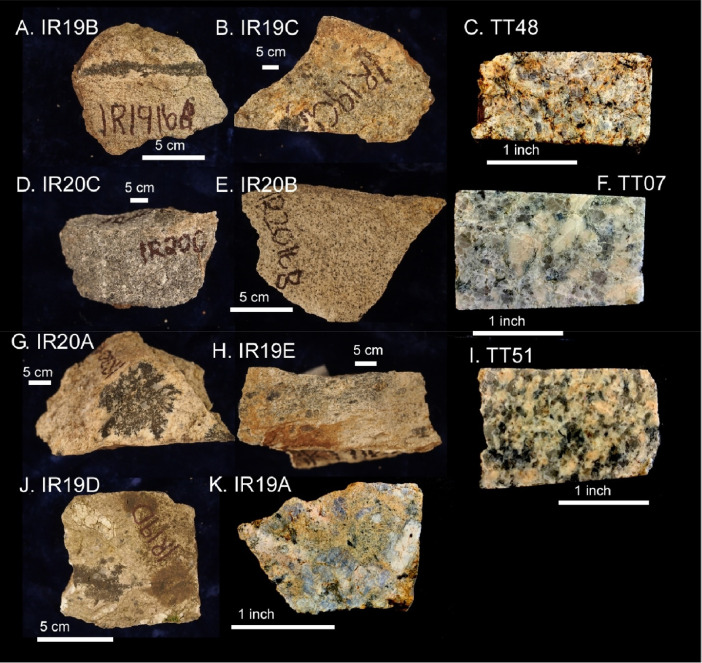
Photographs of the Gemeric and Veporic unit granite assemblages that were analysed in this study. Betliar granites are shown in panels (A), (B), (D), (E), (G), (H), (J), and (K). Panel (C) is of the Klenovec granite, sampled from the Veporic unit. Panel (F) is from the Elisabeth Mine, whereas panel (I) is from the Hnilec granite.

**Fig. 2:**
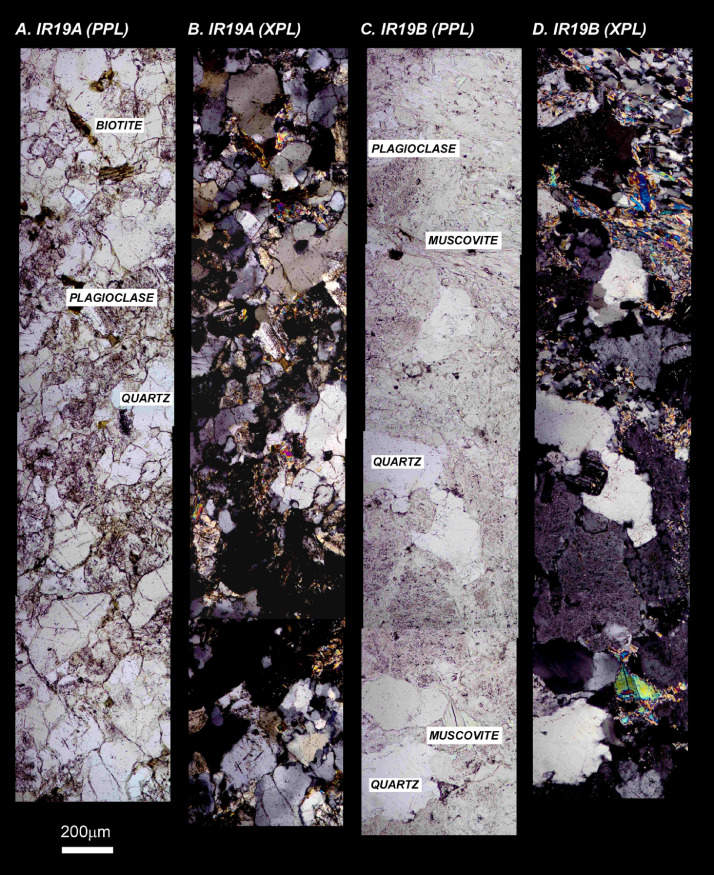
Petrographic images of Betliar granite samples (A) IR19A (plane-polarized light), (B) IR19A (crossed-polarized light), (C) IR19B (plane-polarized light), (D) IR19B (crossed-polarized light). Some minerals are indicated. The plane and cross-polarized images are from the same approximate area.

**Fig. 3:**
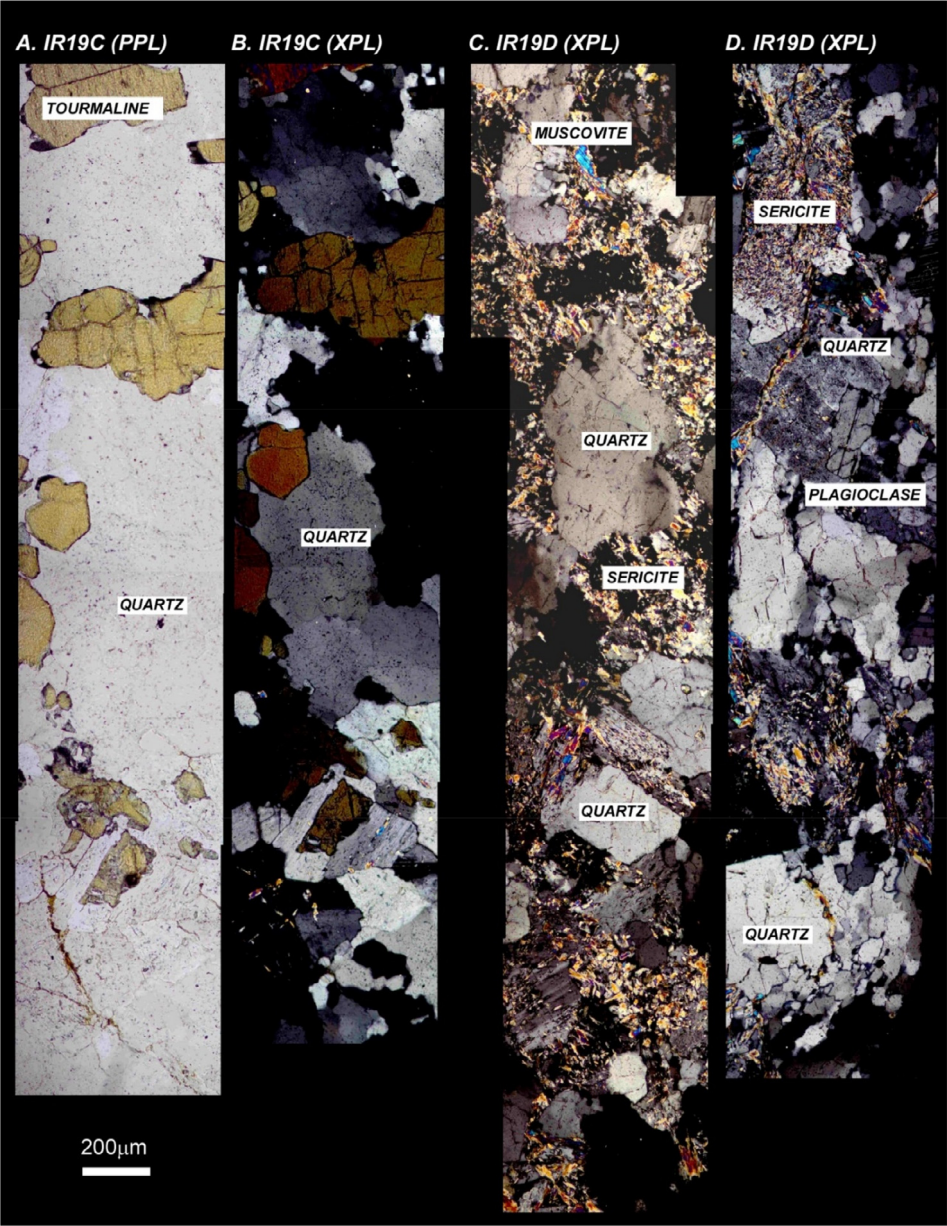
Petrographic images of Betliar granite samples (A) IR19C (plane-polarized light) (B) IR19C (crossed-polarized light), (C) IR19D (crossed-polarized light), and (D) IR19D (crossed polarized light). Some minerals are indicated. The plane- and cross-polarized images in sample IR19C are from the same approximate area.

**Fig. 4:**
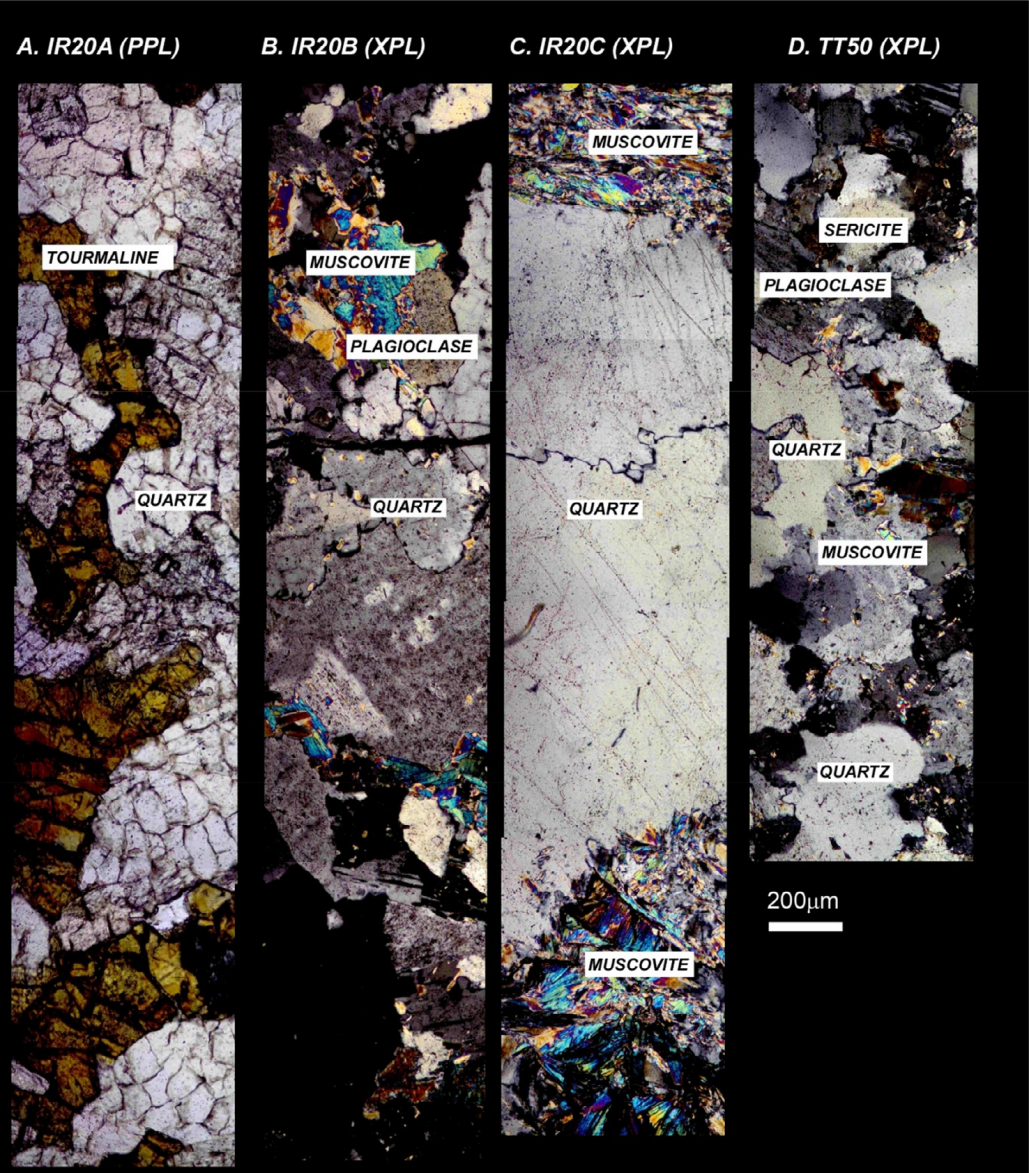
Petrographic images of Betliar granite samples (A) IR20A, (B) IR20B, and (C) IR20C. Panel D shows Hnilec granite sample TT51. All images are taken in crossed-polarized light, except (A) IR20A. Some minerals are indicated.

**Fig. 5:**
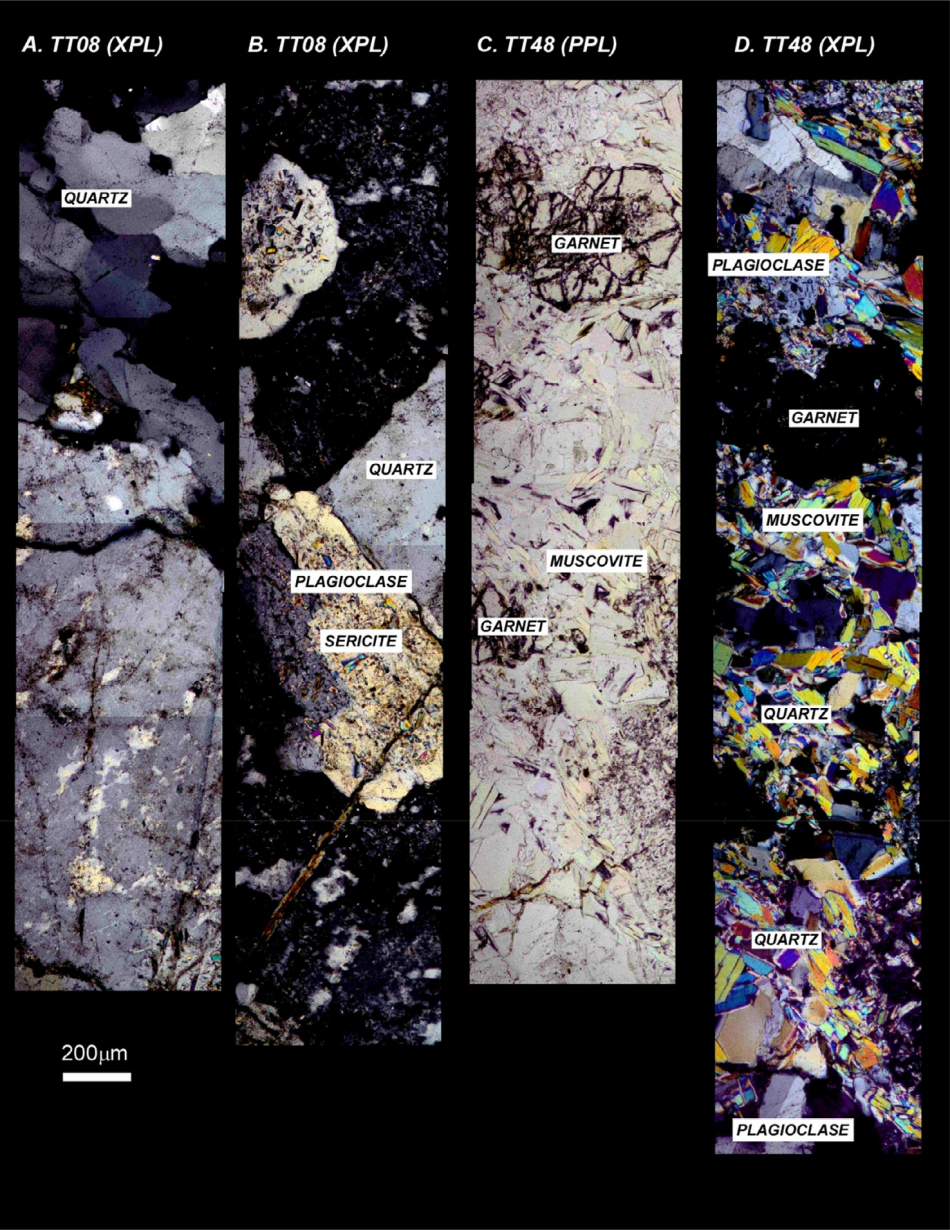
Panels (A) and (B) are petrographic images of Elisabeth Mine granite sample TT08 in crossed-polarized light. Panels (C) and (D) show petrographic images of Klenovec granite sample TT48. Panel (C) is in plane-polarized light, whereas panel (D) is in crossed-polarized light. Some minerals are indicated.

**Fig. 6:**
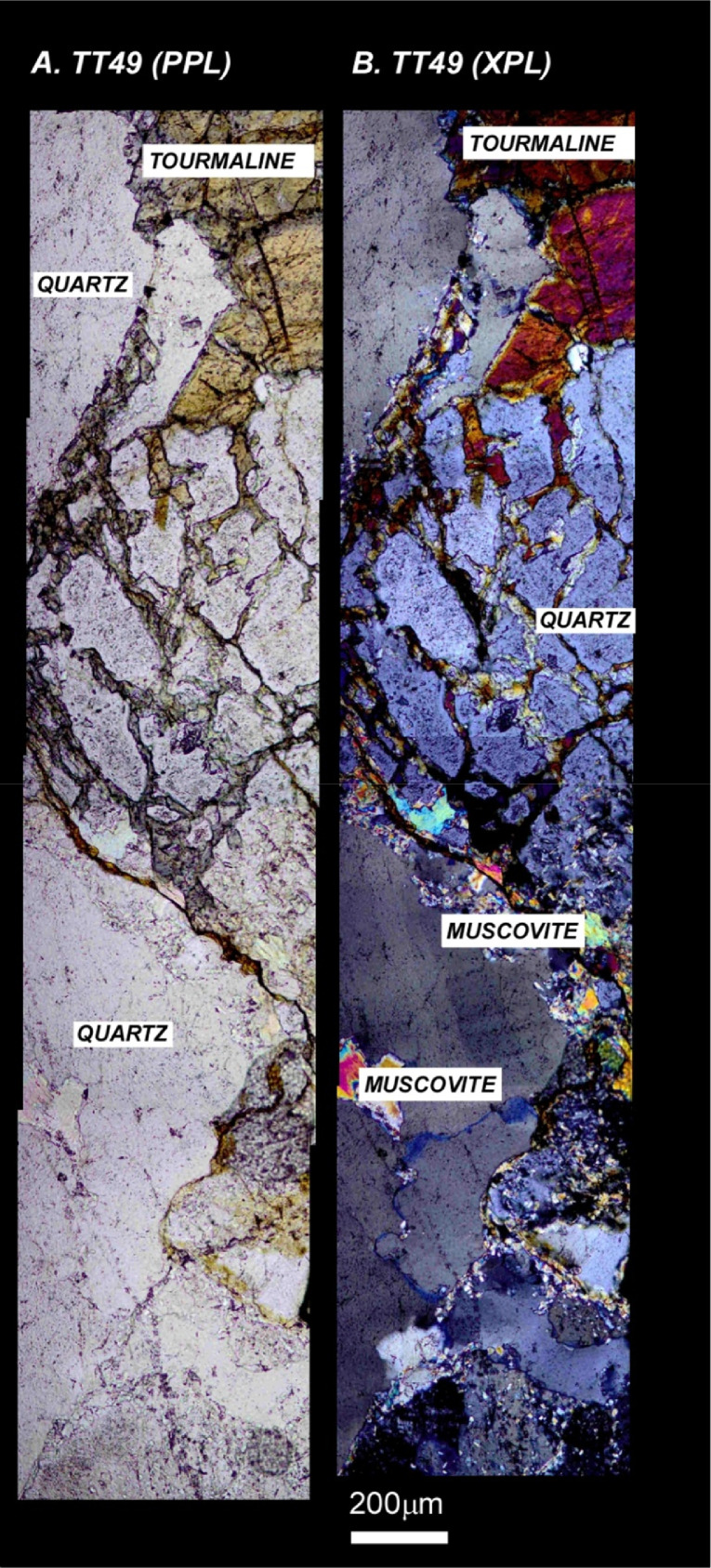
Panels (A) and (B) are petrographic images of Poproč granite sample TT49. Panel (A) is in plane-polarized light, whereas panel (B) is in crossed-polarized light. The images are taken of the same approximate area. Some minerals are indicated.

**Fig. 7:**
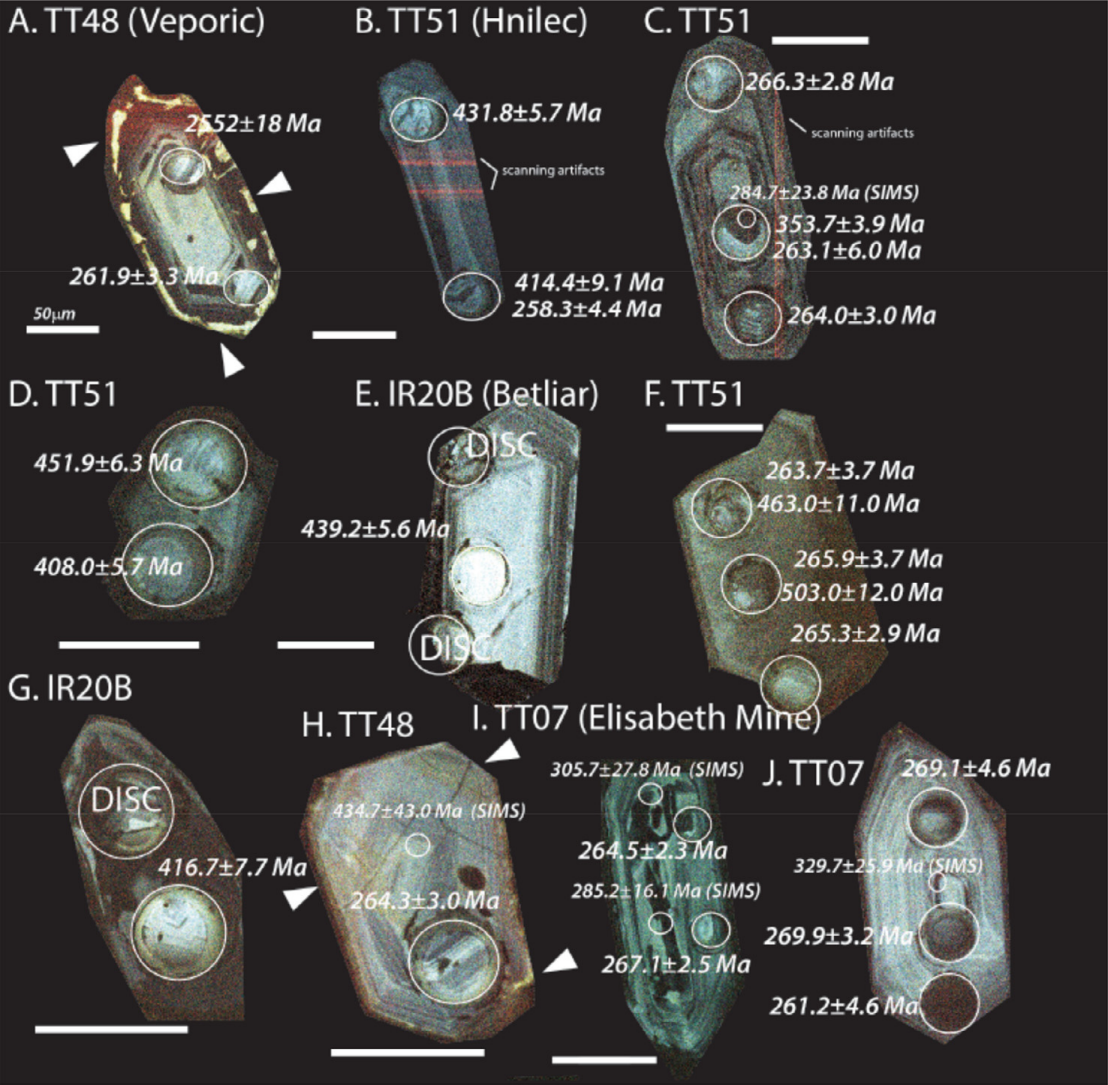
Color CL images of selected Gemeric and Veporic granite zircons with ages older than the Permian. Panels indicate the sample numbers, and the scale bar is 50µm. All ages are LA-ICP-MS (±2σ), except for panels (C) TT51 and (H) TT48, which indicate SIMS ages (±1σ) in smaller font and with a smaller spot. The dated spots are circled in each panel. DISC= discordant age and is not reported. Arrowheads indicate the locations of brighter yellow CL zones on the edges of zircon from TT48 sample, which is a characteristic of zircons from this sample. Note that some of the red lines seen in zircons in panels B and C are an artifact of the scanning process. Panels A, F, and H are published also in [10].

**Fig. 8:**
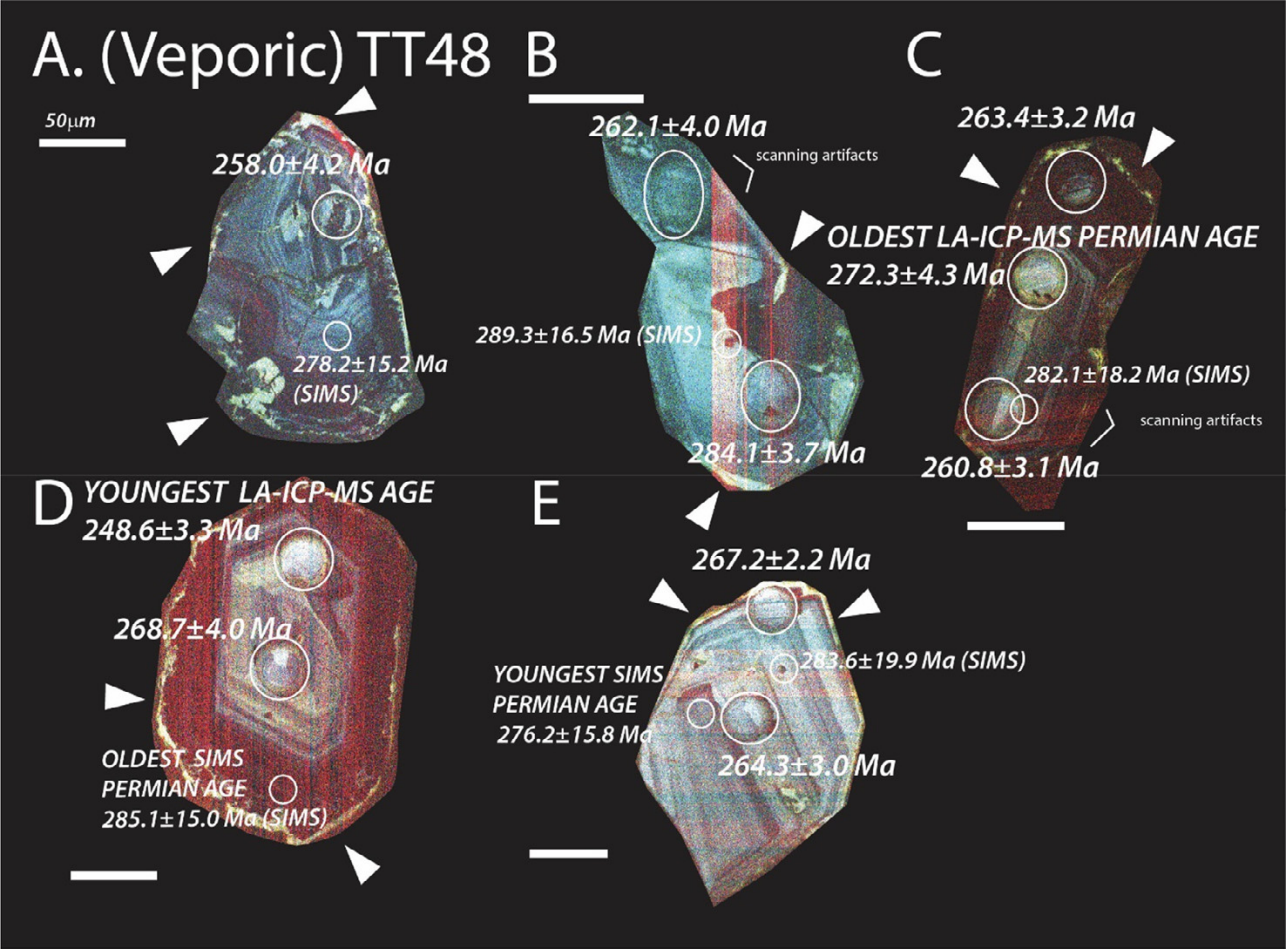
Color CL images of selected zircons from Veporic unit sample TT48. All ages are LA-ICP-MS (±2σ), except otherwise indicated by a SIMS age (±1σ) in smaller font and with a smaller spot. The dated spots are circled in each panel. Arrowheads indicate the locations of brighter yellow CL zones on the edges of zircon from this sample. Some of the faint red lines seen in some of the zircons are an artifact of the scanning process. Panels C, D, and E are published also in [10].

**Fig. 9:**
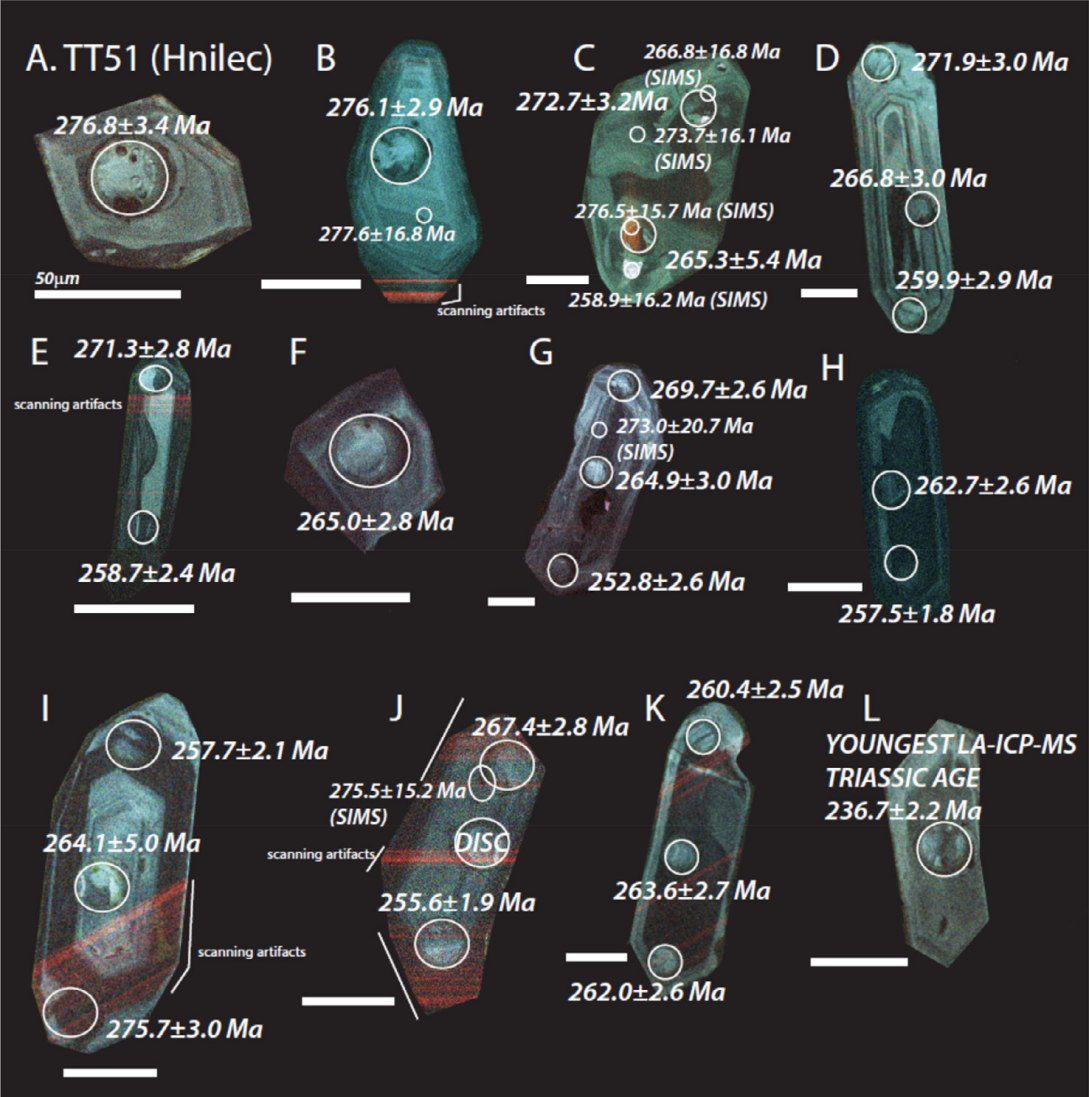
Color CL images of selected zircons from Hnilec granite sample TT51. All ages are LA-ICP-MS (±2σ), except otherwise indicated by a SIMS age (±1σ) in smaller font and with a smaller spot. The dated spots are circled in each panel. Some of the faint red lines seen in some of the zircons are an artifact of the scanning process. Panels B, F, K, and L are published also in [10].

**Fig. 10:**
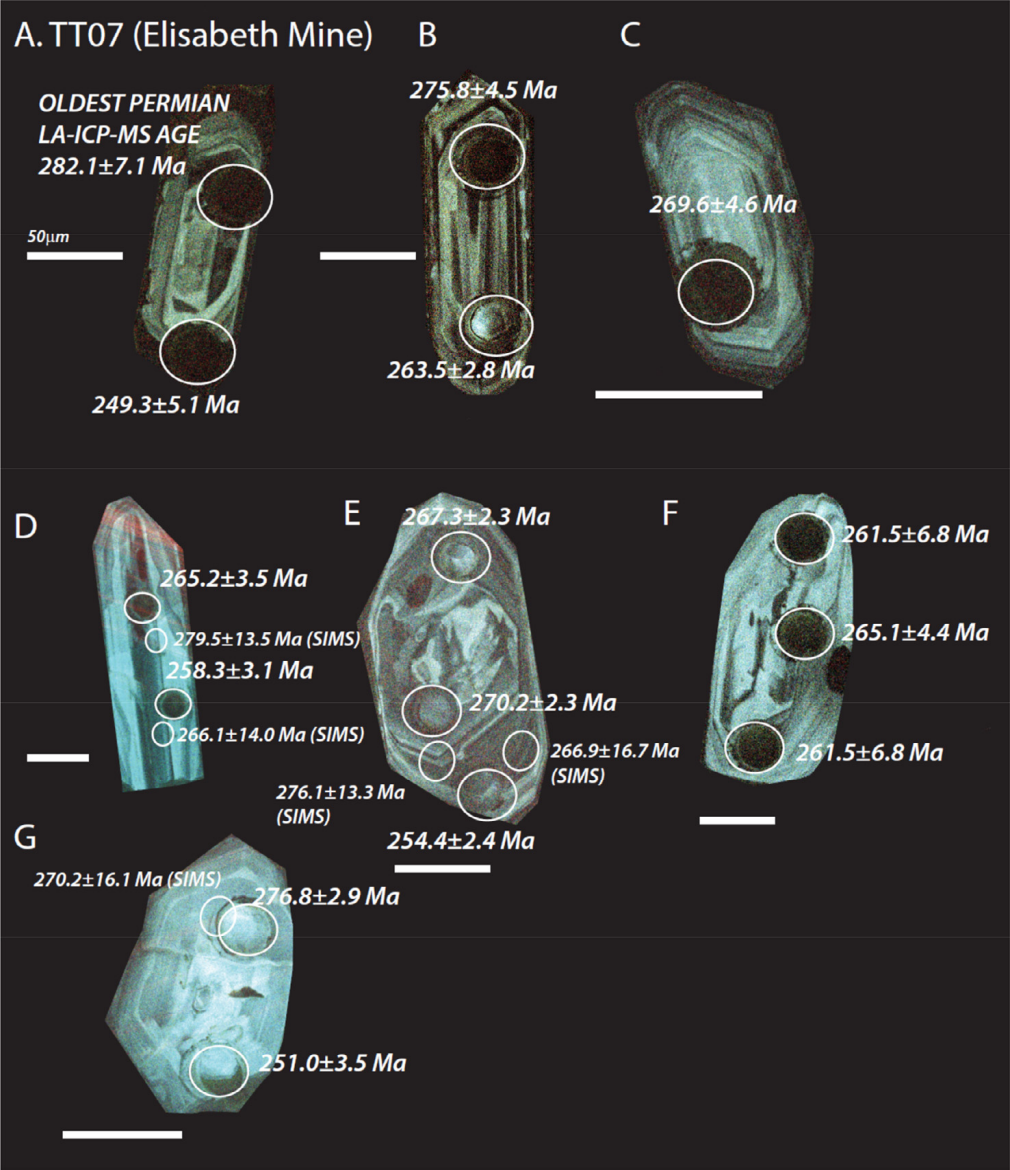
Color CL images of selected zircons from Elisabeth Mine sample TT07. All ages are LA-ICP-MS (±2σ), except otherwise indicated by a SIMS age (±1σ) in smaller font and with a smaller spot. The dated spots are circled in each panel. Panels A, B, D, and E are published also in [10].

**Fig. 11:**
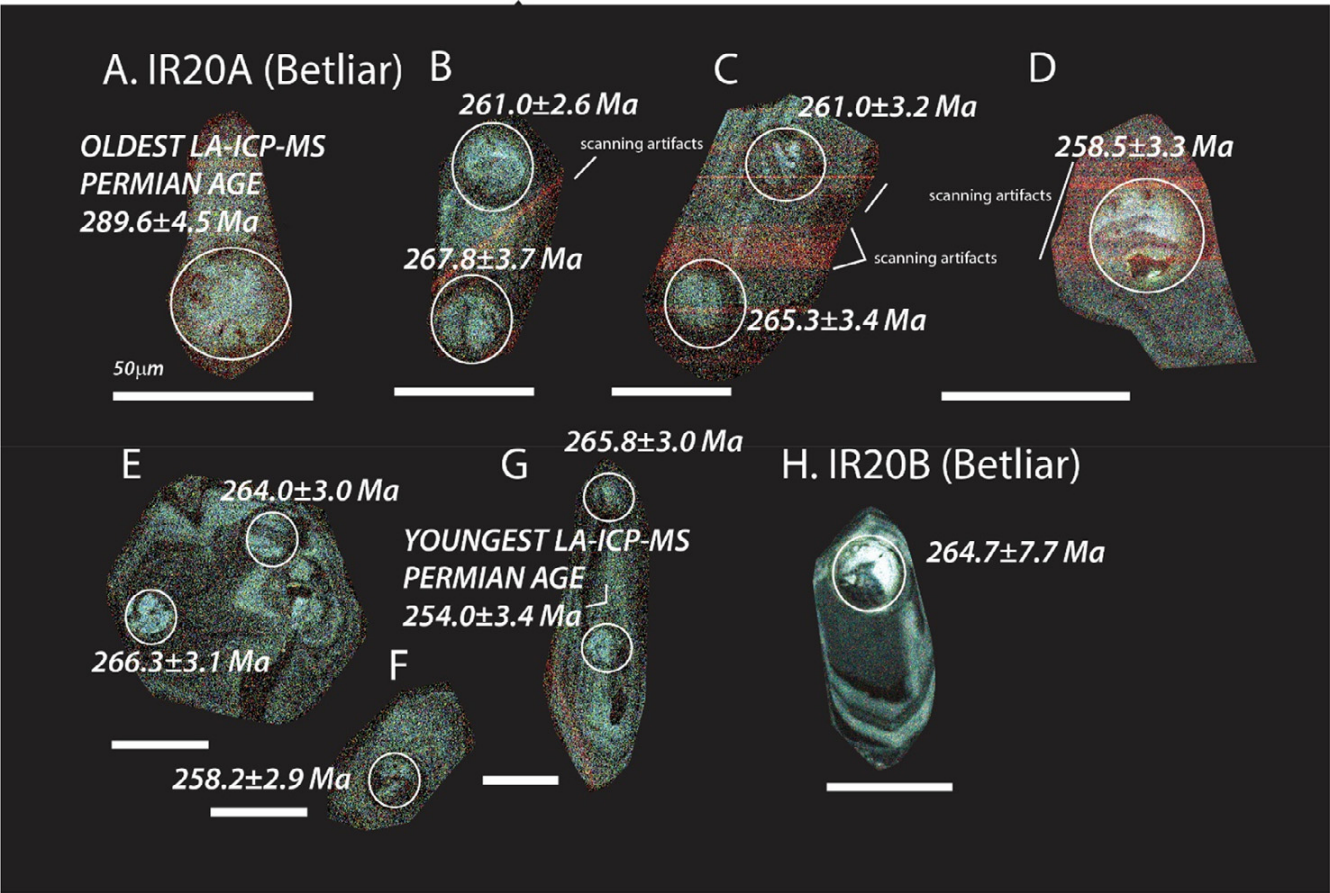
Color CL images of selected zircons from Betliar granite sample TT20A and B. All ages are LA-ICP-MS (±2σ). The dated spots are circled in each panel. Some of the faint red lines seen in some of the zircons are an artifact of the scanning process. Panels A, E, F, G, and H are published also in [10].

**Fig. 12:**
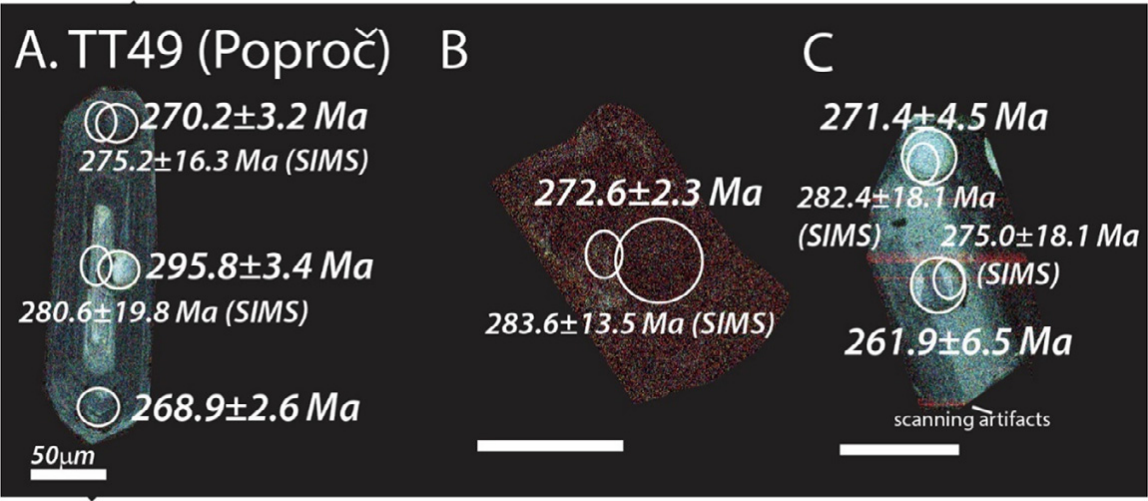
Color CL images of selected zircons from Poproč granite sample TT49. All ages are LA-ICP-MS (±2σ), except otherwise indicated by a SIMS age (±1σ) in smaller font and with a smaller spot. The dated spots are circled in each panel. Some of the faint red lines seen in some of the zircons are an artifact of the scanning process.

The next two tables provide major and trace element geochemistry from the Betliar and Klenovec granite assemblages.

**Fig. 13:**
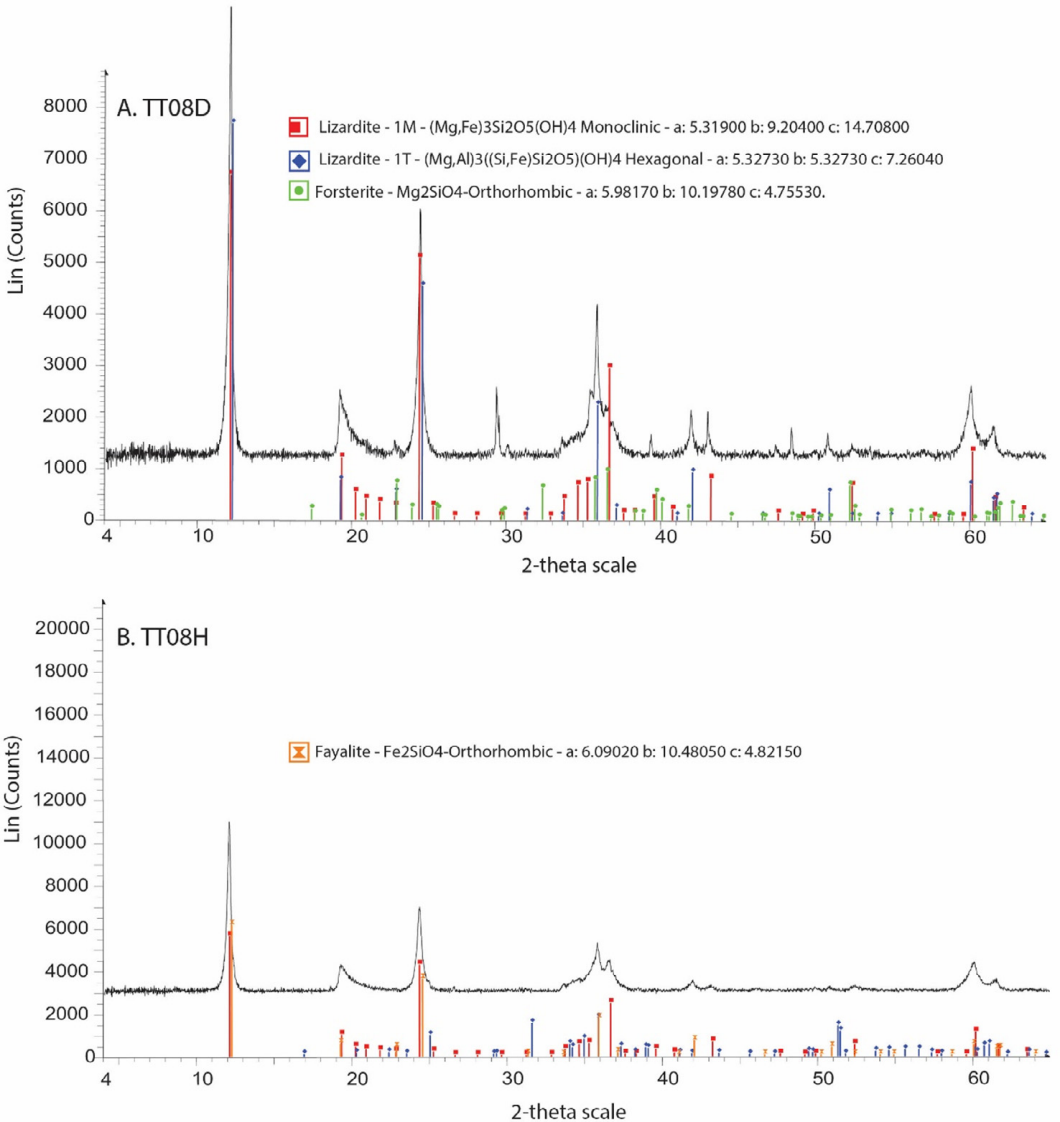
X-ray Diffraction (XRD) data from serpentinized samples collected at the TT08 locality, indicating lizardite, forsterite, and fayalite. The 2-theta scale extends from 4° to 65.007° with 0.02° increments steps. The total step time was 192 s and the data was collected at room temperature (25 °C). (A) Red lines correspond to lizardite 1 M [(Mg,Fe)_3_Si_2_O_5_(OH)_4_] at 31.19%. Crystal parameters are labeled in the figure, with α = 90.0, β = 96.9, and γ = 90.0. The symmetry is base-centered. Blue lines correspond to lizardite 1T [(Mg,Fe)_3_Si_2_O_5_(OH)_4_] at 35.86%. Crystal parameters are labeled in the figure, with α = 90.0, β = 90.0, and γ = 120.0. The symmetry is primitive. Green lines correspond to forsterite, syn - Mg_2_SiO_4_ at 4.32%. Crystal parameters are labeled in the figure, with α = 90.0, β = 90.0, and γ = 90.0. The symmetry is base-centered. In panel (B), red lines correspond to lizardite 1 M [(Mg,Fe)_3_Si_2_O_5_(OH)_4_] at 26.34%. Crystal parameters are labeled in the figure, with α = 90.0, β = 96.9, and γ = 90.0. The symmetry is base-centered. Blue lines correspond to fayalite, syn Fe2+2SiO4 at 8.48% Crystal parameters are labeled in the figure, with α = 90.0, β = 90.0, and γ = 90.0. The symmetry is primitive. Orange lines correspond to lizardite 1T [(Mg,Fe)_3_Si_2_O_5_(OH)_4_] at 28.8%. Crystal parameters are labeled in the figure, with α = 90.0, β = 90.0, and γ = 120.0. The symmetry is primitive.

Fig. 13 presents XRD data from two samples collected from altered serpentinite samples TT08D and TT08H.

Details regarding the geochronological data from the zircons grains dated in this study are available as Excel Spreadsheets.

Table 4. Excel spreadsheet of the zircon geochronological data obtained using SIMS.

Table 5. Excel spreadsheet of the zircon geochronological data obtained using LA-ICP-MS.

## 2 Experimental Design, Materials, and Methods

### 2.1 Geochemistry

Eight individual portions of Betliar pluton (IR19A-E and IR20A-C) and seven from the Klenovec granite (VZ-3/40 m, VZ-3/93 m, VZ-4/118 m, VZ-4/142 m, LH-39/04, LH 4/04, and LH 72/04) were analyzed for major and trace elements (Table 1, Table 2, Table 3). Fusion Inductively Coupled Plasma (ICP) spectrometry was applied to fused samples using a Perkin–Elmer Sciex ICP-Mass Spectrometer (MS) to generate all major elements and *Sc*, Be, V, Ba, Sr, Y, Zr. Fusion ICP-MS was used for all other elements. Three blanks and five controls (three before and two after) were analyzed per group of samples. Duplicates were fused and analyzed every 15 samples, and the instrument was recalibrated after every 40 analyses. Betliar samples were analyzed at Activation Laboratories in Canada, whereas those from the Veporic Unit were obtained from the Laboratory State Geological Institute of Dionýz Štúr, Spišská Nová Ves, in Slovakia.

**Table 3:** Summary of the whole-rock geochemical analysis of samples from the Klenovec Pluton.

Analyte Symbol	VZ-3/40m	VZ-3/93m	VZ-4/118m	VZ-4/142m	LH-39/04	LH-4/04	LH-72/04
SiO_2_	70.08	76.33	71.34	75.49	73.18	71.64	71.59
Al_2_O_3_	14.39	13.77	14.69	13.64	14.23	14.96	15.19
Fe_2_O_3_(T)	2.260	0.530	2.010	0.430	1.870	2.240	2.570
Fe_2_O_3 (calc)_	0.782	0.286	0.654	0.186	0.592	0.417	0.570
FeO	1.330	0.220	1.220	0.220	1.150	1.640	1.800
MnO	0.740	0.200	0.730	0.230	0.460	0.037	0.064
MgO	2.090	0.430	1.760	0.620	0.760	0.730	0.780
CaO	0.070	0.015	0.064	0.018	0.059	1.240	0.480
Na_2_O	3.91	4.35	3.68	3.68	3.04	3.31	4.28
K_2_O	3.04	3.12	3.78	4.53	4.85	4.08	3.11
TiO_2_	0.310	0.039	0.300	0.078	0.265	0.309	0.307
P_2_O_5_	0.170	0.180	0.160	0.160	0.160	0.180	0.190
Stotal	0.020	0.070	0.020	0.050	0.020	0.040	0.050
SO_3_	0.060	0.170	0.030	0.130	0.030	0.040	0.040
F	0.150	0.050	0.140	0.050	0.090	0.070	0.170
H_2_O+	0.300	0.280	0.390	0.250	0.330	0.250	0.160
H_2_O-	0.340	0.290	0.230	0.270	0.310	0.290	0.290
CO_2_	1.280	0.030	0.190	0.180	<0.01	<0.04	<0.04

Total	101.3	100.7	101.4	100.2	101.4	101.5	101.6

Be	3.5	5.4	3.2	8.2	2.5	5.6	3.9
V	23	<5	16	5	12	25	24
Ba	236	153	317	150	348	329	301
Sr	91	43	135	49	67	119	71
Y	14	5	15	9	23	15	16
Zr	131	21	125	35	156	116	131
Cr	11	6	11	8	12	n.m	n.m
Co	3	1	<1	2	2	3	4
Ni	4	2	6	4	6	15	14
Cu	5	6	<2	10	3	2	<2
Zn	61	17	50	13	41	41	66
Ga	18	21	17	18	20	18	17
As	3	4	6	5	210	9	30
Rb	203	176	202	194	310	200	209
Nb	12	11	13	11	19	12	12
Sn	16	19	13	11	15	9	15
Cs	12	9	13	7	15	10	13
La	23	3	25	6	37	26	23
Ce	42	2	43	9	72	47	42
Pr	4.6	0.8	4.9	1.6	6.8	4.8	4.9
Nd	16.6	2.1	17.6	4.9	25.1	17	17
Sm	3.9	0.9	4	1.6	5.6	3.8	4
Eu	0.64	0.06	0.66	0.11	0.5	0.6	0.7
Gd	3.3	0.8	3.5	1.4	4.9	2.9	3.1
Tb	0.5	0.2	0.6	0.3	0.8	0.5	0.5
Dy	2.7	1	2.8	1.7	4.1	2.5	2.8
Ho	0.5	0.2	0.5	0.3	0.7	0.5	0.5
Er	1.2	0.5	1.2	0.9	1.9	1.4	1.6
Tm	<0.1	<0.1	<0.1	<0.1	0.2	<0.1	0.1
Yb	1.1	0.6	1.2	1.1	1.8	1.1	1.3
Lu	0.22	0.07	0.18	0.17	0.34	0.17	0.22
W	<10	<10	10	<10	10	<10	12
Pb	9	13	20	20	29	29	11
Bi	<0.1	1.2	0.3	0.2	0.4	0.8	<0.1
Th	7	<3	10	4	19	n.m.	n.m.
B	18	16	14	9	17	16	13
Hg	0.03	0.04	0.03	0.05	0.04	<0.01	<0.01
Li	47	14	32	9	37	33	59

Measured but below detection limits: Cl <0.01 ppm, Ag <0.4 ppm, Mo < 3 ppm; Sb<2 ppm; n.m. = not measured. Samples VZ-3/40 m. VZ-4/118 m, and LH-39/04, and LH 72/04a are porphyry-type granites, VZ-3/93 m is a pale muscovite granite, and samples VZ-4/142 m and LH 4/04 are fine-grained leucocratic granites.

### 2.2 X-ray diffraction

X-ray Diffraction (XRD) data were obtained using a Bruker D8 Advance, which provides routine, qualitative mineral identification in rock powders. We analyzed powdered serpentinite assemblages TT08D and TT08H. Samples for XRD were carefully ground rock powders. Data were interpreted using automation software with integrated pattern analysis by Bruker EVA and Topas using the International centre for Diffraction Data (ICDD) Powder Diffraction File-2 (PDF-2).

### 2.3 Geochronology

Granite samples TT51 (Hnilec), IR19A and B, and IR20A and B (Betliar), TT07 (Elisabeth Mine), TT49 (Poproč), and TT48 (Veporic, Klenovec), and sedimentary rock sample TT08 were subjected to common mineral separation techniques to extract zircon grains (150–200 μm in length). All were examined optically during the mounting process to select euhedral grains and eliminate the analysis of cracked or metamict zircons. Zircons from samples IR19A and B from Betliar Pluton were mounted on double-sided tape, and whole grains were dated using an Element2 High Resolution (HR)-ICP-MS with an Excimer (192 nm) laser ablation system instrumentation in the Geo-Thermochronometry lab at the University of Texas at Austin. LA-ICP-MS analytical procedures are similar to [1]. All other zircons were mounted in epoxy with a set of AS3 zircon reference (1099.1 ± 0.5 Ma) [2] and polished to expose cross-sections for further imaging using cathodoluminescence (CL). These zircons were dated using both LA-ICP-MS and secondary ion mass spectrometry (SIMS) using a CAMECA IMS 1280-HR at UCLA. For SIMS analysis, we also used secondary age standards R33 (419 Ma) [3], Plešovice (337 Ma) [4], and zircon U/Th standard 91500 (1065±0.3 Ma, U = 81.2 ppm, Th = 28.6 ppm [5]). The use of standard 91500 allows an estimation of zircon spot U and Th contents. For LA-ICP-MS analysis, elemental and isotopic fractionation of Pb/U and Pb isotopes, respectively, is corrected by interspersed analysis of primary and secondary zircon standards with a known age (GJ1) [6] and Pak1, an internal age standard. Excel Tables provide details of the geochronological data (see Texas Scholar Works, University of Texas Libraries, direct URL to data: https://doi.org/10.18738/T8/PFWPNR).

Before SIMS analysis, mounts were cleaned in Ethylenediaminetetraacetic acid (EDTA) disodium salt dehydrate (C_10_H_14_N_2_Na_2_O_8_·2H_2_O) followed by methanol and distilled water to reduce the potential for common Pb contamination [7], followed by coating in gold. An oxygen beam (~20 µm spot size) sputtered isotopes of U, Th, and Pb from the surface to a depth of <5 µm on the zircon grain. Given the small amount of sample consumed, the approach is minimally destructive and allows for future analysis of these grains by LA-ICP-MS.

During SIMS analysis, a 10–15 nA ^16^O primary beam focused to a spot 10–15 µm diameter to generate +10 kV secondary ions. The mass resolution was set to ~7000, and oxygen flooding was applied to increase Pb+ yields. A 30 s pre-sputtering time allowed the removal of potential surficial contamination. For each analysis, secondary ion intensities were acquired in nine magnet cycles through the species ^94^Zr_2_^16^O, ^204^Pb, ^206^Pb, ^207^Pb, ^208^Pb, ^232^Th, ^238^U, and ^238^U^16^O. Zircon standards R33 (*n* = 24), 91,500 (*n* = 3), and Plešovice (*n* = 6) were run initially, followed by AS3 grains (*n* = 47). The AS3 was also analyzed after every five to six unknown spots. A calibration curve of UO+/*U*+ = 1.561(Pb+/*U*+, Relative Sensitivity Factor) + 4.229±0.087 reproduced the ^238^U-^206^Pb age of AS3 to 1103±56 Ma (±1σ). Standard 91,500 yields a ^238^U-^206^Pb age of 1077.3 ± 62.0 Ma. Twenty-four spots on standard R33 yield 419.5 ± 24.1 Ma, and six on Plešovice yield 329.9 ± 18.4 Ma. The age uncertainty is an estimate for analytical precision. The UO+/*U*+ values sputtered from the AS3 grains average 6.448±0.034, with a range of 6.046±0.040 to 6.879±0.087. Ideally, the unknown lies between those values for the best precision. All analyses on reference zircon AS3 were reduced using a common ^204^Pb correction, whereas the unknown grains were subjected to ^208^Pb corrections. SIMS data reduction, Concordia diagrams, and age calculations were performed using the software package ZIPS (v3.1.1; Chris Coath, University of Bristol). Common Pb corrections were applied using the evolution model of [8] and decay constants and ratios recommended by [9]. Uncertainties of the decay constants are included in all U-Pb ages. All SIMS ages discussed in the text are ^238^U-^206^Pb ages and are reported with ±1σ uncertainty.

Due to the larger uncertainty in the SIMS ages, we applied LA-ICP-MS geochronology to generate higher-precision results from more zircons grains. In this approach, an ablated dry aerosol is introduced into the HR-ICP-MS using ultra-high purity He carrier gas for ^238^U–^232^Th and ^206^Pb–^208^Pb isotopic measurements using ion-counting. Each analysis consisted of a two-pulse cleaning ablation, a background measurement taken with the laser off, a 30 s measurement with the laser firing, and a 30 s cleaning cycle. The laser spot size used 30 µm. Common Pb was corrected using the measured ^204^Pb (Hg-corrected) and assuming the initial composition reported by [8]. The unknown to standard measurement ratio was generally 3:1 or 4:1. Uncertainty resulting from calibration correction is generally 1–2% for both ^206^Pb/^207^Pb and ^206^Pb/^238^U. We report the ^238^U-^206^Pb age if the zircon was less than 850 Ma and the ^207^Pb-^206^Pb age if the grain was older than 850 Ma. We selected data used for age calculations using time-resolved isotope ratio traces during ablation to limit the possibility of mixed ages, as changes in U and Pb isotopic values could be detected as the laser penetrated the grain. Ages were filtered for concordance, and all are presented in the Excel files.

## Ethics Statement

This presents original research. When we have used the work of others, this has been appropriately cited or quoted.

## CRediT Author Statement

**Gabriel Villaseñor:** Writing - Original draft preparation, Data generation; **Elizabeth J. Catlos:** Conceptualization, Methodology, Data curation, Writing - Original draft preparation, Supervision; **Igor Broska:** Conceptualization, Writing - Reviewing and Editing; **Milan Kohút:** Conceptualization, Writing - Reviewing and Editing; **Ľubomír Hraško:** Data generation, Writing - Reviewing and Editing; **Kimberly Aguilera:** Data generation, Writing - Reviewing and Editing; **Thomas M. Etzel:** Writing - Reviewing and Editing; **J. Richard Kyle:** Writing - Reviewing and Editing; **Daniel Stockli:** Methodology, Data generation, Data curation, Writing - Reviewing and Editing.

## Declaration of Competing Interest

The authors declare that they have no known competing financial interests or personal relationships which have or could be perceived to have influenced the work reported in this article.

## Appendix Supplementary materials

Image, application 1

Image, application 2
